# Automated Selection of Hotspots (ASH): enhanced automated segmentation and adaptive step finding for Ki67 hotspot detection in adrenal cortical cancer

**DOI:** 10.1186/s13000-014-0216-6

**Published:** 2014-11-25

**Authors:** Hao Lu, Thomas G Papathomas, David van Zessen, Ivo Palli, Ronald R de Krijger, Peter J van der Spek, Winand NM Dinjens, Andrew P Stubbs

**Affiliations:** Department of Bioinformatics, Erasmus MC, University Medical Center, PO Box 2040, 3000 CA Rotterdam, The Netherlands; Department of Pathology, Josephine Nefkens Institute, Erasmus MC, University Medical Center, PO Box 2040, 3000 CA Rotterdam, The Netherlands; Department of Pathology, Reinier de Graaf Hospital, Delft, The Netherlands

**Keywords:** Adrenal Cortical Carcinoma (ACC), Gastroenteropancreatic Neuroendocrine Tumor (GEP-NET), Automated Selection of Hotspots (ASH), Adaptive step finding, Ki67 LI, ImmunoRatio, Galaxy, Virtual machine (VM)

## Abstract

**Background:**

In prognosis and therapeutics of adrenal cortical carcinoma (ACC), the selection of the most active areas in proliferative rate (hotspots) within a slide and objective quantification of immunohistochemical Ki67 Labelling Index (LI) are of critical importance. In addition to intratumoral heterogeneity in proliferative rate i.e. levels of Ki67 expression within a given ACC, lack of uniformity and reproducibility in the method of quantification of Ki67 LI may confound an accurate assessment of Ki67 LI.

**Results:**

We have implemented an open source toolset, Automated Selection of Hotspots (ASH), for automated hotspot detection and quantification of Ki67 LI. ASH utilizes NanoZoomer Digital Pathology Image (NDPI) splitter to convert the specific NDPI format digital slide scanned from the Hamamatsu instrument into a conventional tiff or jpeg format image for automated segmentation and adaptive step finding hotspots detection algorithm. Quantitative hotspot ranking is provided by the functionality from the open source application ImmunoRatio as part of the ASH protocol. The output is a ranked set of hotspots with concomitant quantitative values based on whole slide ranking.

**Conclusion:**

We have implemented an open source automated detection quantitative ranking of hotspots to support histopathologists in selecting the ‘hottest’ hotspot areas in adrenocortical carcinoma. To provide wider community easy access to ASH we implemented a Galaxy virtual machine (VM) of ASH which is available from http://bioinformatics.erasmusmc.nl/wiki/Automated_Selection_of_Hotspots.

**Virtual Slides:**

The virtual slide(s) for this article can be found here: http://www.diagnosticpathology.diagnomx.eu/vs/13000_2014_216

## Background

Adrenal cortical carcinoma (ACC) is a rare type of endocrine malignancy with an estimated incidence of 0.7–2.0 cases per million population per year and a poor overall prognosis [1]. According to recent evidence from the European Network for the Study of Adrenal Tumors (ENS@T) ACC study group, the resection status and the Ki67 labelling index (LI) in both localized and advanced ACC [2,3] constitute the most relevant prognostic parameters [4]. In this regard, it has been suggested that the histopathology report should include Ki67 LI along with confirmation of the adrenocortical origin on immunohistochemical grounds, Weiss score and resection status [4]. Importantly, Ki67 LI has been integrated in treatment flow charts for ACC patients with either tumor amenable to radical resection or advanced disease [4].

Taken together, the production of accurate and reproducible Ki67 LIs remains a key issue and main responsibility of pathologists. It should be recognized that various factors, such as pre-analytical, analytical, interpretation, scoring, and data analysis, might affect Ki67 LI [5]. Given the biological heterogeneity of Ki67 immunostaining across tumor specimens [5,6], the area of slide read has been controversial for Ki67 LI assessment e.g. in breast cancer [5,7]. According to the European Society of Neuroendocrine Tumors (ENETS), the mitotic count and the Ki67 LI should be assessed in areas with the highest proliferating activity (hotspots) in order to determine the proliferation grade in gastroenteropancreatic neuroendocrine tumors (GEP-NETs) [8]. As far as ACCs are concerned, there is not only lack of studies addressing the issues of a potential biological heterogeneity of Ki67 staining and inter-observer variation, but also different methods of objective quantification of the Ki67 proliferative index.

In routine diagnostic practice, representative areas of slides are manually selected by histopathologists using visual examination of whole mount Ki67-immuostained slides at a low magnification. Of note, this process might lack reproducibility and affect the Ki67 LI [5]. Since digitized immunohistochemical (IHC) stained tissue sections have become amenable to the application of computerized image analyses, two independent groups have developed either a hybrid clustering approach for the detection of Ki67 hotspots in whole tumor slide images [9] or a simplified computerized method for hotspot detection in digitized IHC slides [10]. In this context, we developed Automated Selection of Hotspots (ASH) to provide clinical labs with the ability to determine the most active areas in proliferative rate within a slide and subsequently quantitate Ki67 LI using a desktop PC without requiring extensive bioinformatics support. ASH uses Galaxy [11] as a simple graphical user interface and to join the components of ASH into an analytical workflow for hotspot detection and this, Galaxy is contained in a VMware virtual machine (VM) [12] which ensures that the system is platform independent. The use of VM technology has been highlighted by *Nocq* et al. [13], to improve the usability of next generation sequencing software by simply sharing entire installations.

We believe that this is the first time that Galaxy-VM has been used to deliver single user (on a personal computer) or as a multi-user (on a server) hotspot detection software with the same easy access via the Galaxy graphical user interface (GUI).

## Method

**Figure 1:**
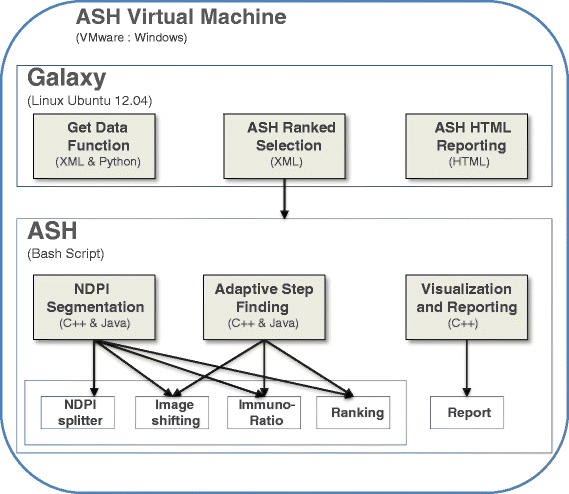
**Software architecture overview: the ASH virtual machine contains both the ASH image analysis and the graphical user interface provided by Galaxy.** ASH image analysis, NDPI segmentation an Adaptive Step finding components use three of the same methods.

**Figure 2:**
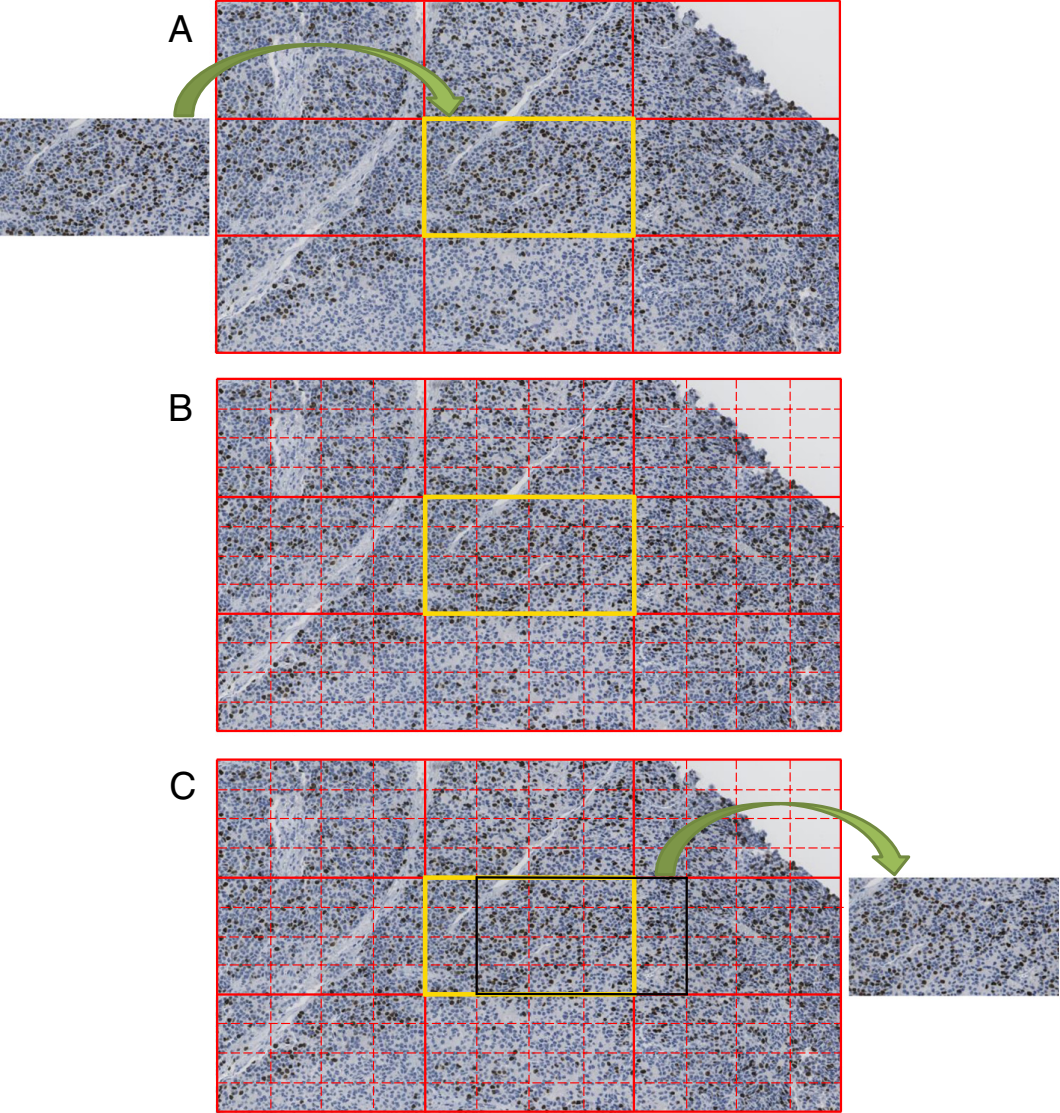
**Adaptive step finding.** This figure represents ¼ step shift analysis; **(A)** Eight neighboring images are generated around each of the top ten ImmunoRatio images (left hand side) obtained from the segmentation step of ASH; **(B)** this 3 × 3 image is divided into totally 81 image blocks by step shifting ¼ and the ImmunoRatio is for each block; **(C)** image with highest ImmunoRatio among these 81 images is outlined (black) and displayed on the right hand side.

ASH is delivered as a virtual machine which consists of 3 classes: NDPI Segmentation, Adaptive Step Finding and Reporting Visualization (Figure 1). NDPI Segmentation used previously described NDPI splitter [14] to split the input image files into A × B matrix followed by a step shift of ^1^/_2_ split image and quantitation Ki67 in all images using ImmunoRatio [15], implemented in ASH. This preprocessing step provides an initial quantitative ranking of all blocks from which the top 10 are used to focus in on the actual ‘hotspot’ fields. To ascertain the exact hotspot positions on the image we develop an Adaptive Step Finding class to adaptively determine the shifting step size, and trade-off between the hotspot detection resolution and system complexity. This Adaptive Step Finding class uses three of the same functions (Image shifting, ImmunoRatio and Ranking) that are used by NDPI Segmentation (Figure 1), however in this class eight blocks are created around the region selected by NDPI Segmentation (Figure 2). The rectangle area is shifted by a step size shrunk 50% every adaptive loop.

ASH provides an end to end workflow for hotspot detection using the functionality of a Galaxy GUI to provide the user with a simple data upload and html style reporting environment.

## Implementation

The application is for digital images obtained on the Hamamatsu NanoZoomer Digital Pathology (NDP) System (Hamamatsu Photonics K.K. Japan), in their proprietary NDP Image (NDPI) file format. NDPI image segmentation using NDPI-splitter is available from [16]. Quantitation of segmented blocks with ImmunoRatio is available from [17]. For image processing, analysis, and visualization, we adopted OpenCV [18]. The ASH software tool is developed on the Ubuntu 12.04 [19] Linux operating system, as a Galaxy application [11] and is distributed as a VMware virtual machine [12] for a Windows user.

The detection of hotspots uses adaptive step finding methodology which has been utilized in engineering for many years [20] and extensively evaluated and validated [21]. Experimental evaluation has demonstrated the effectiveness of the adaptive step size [22] and the adaptive step finding method applied in ASH has the same functionality. The selection of the step size is critical both from the point of view of computational efficiency and detection performance.

To simplify the use of ASH, we have implemented a Galaxy within the same virtual machine (VM) to provide a standardized graphical user interface (GUI) for accessing, running and visualizing ASH. Galaxy is an open, web-based platform [23] and developed tools to upload image files, to analyse the files by ASH in batch mode and to deliver a html report of the selected image with the quantitative ranking of the hotspots displayed in that image. All components and dependencies were created into a VMware virtual machine (VM) [12] which is an environment that is used like any physical computer [24] but also shared by download. The entire virtual machine is usually contained in a few files on the host computer (the physical machine that the virtual machine is running on). This means that all the dependency’s required by ASH, including NDPI splitter, ImmunoRatio, openCV and Galaxy, are replaced by just having VMware installed.

## Results

### Automated selection of hotspots

**Figure 3:**
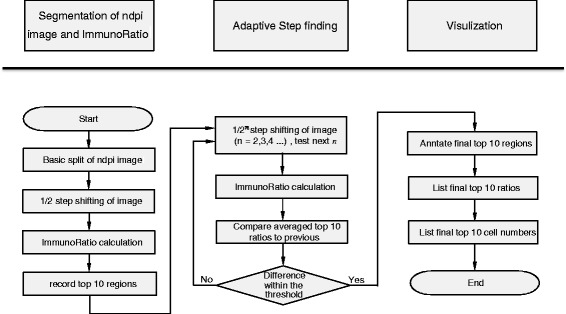
**Image analysis workflow.** The process is divided into the classes, NDPI Segmentation, Adaptive step finding and visual reporting.

The overall work flow for the image analysis outlined in Figure 3 includes the main classes developed for ASH which include NPI Segmentation, Adaptive Step Finding and Visual Reporting.

### NDPI segmentation

**Figure 4:**
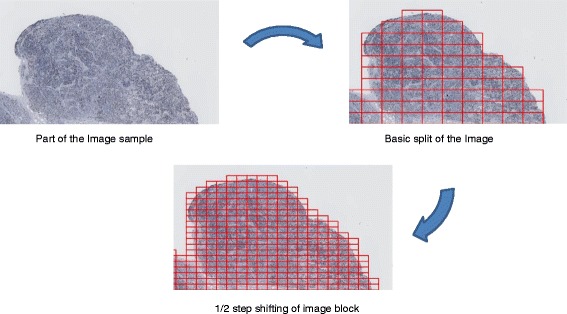
**NDPI Segmentation: the image is segmented using followed by step shifting of these blocks by ½ their size prior to quantitation.**

In the first class, the NDPI Segmentation, the whole digital image scanned from Hamamatsu NanoZoomer is first divided with the NDPI splitter (Figure 3). NDPI splitter processes the basic split of the image from a single (100 K × 100 K pixel) NDPI image into thousands of smaller (2 K × 1 K pixels) images known as image blocks. Step shifting of ½ the size of an image block is performed to provide overlapping blocks, in order to scan more area and improve ImmunoRatio detection resolution. For the primary selection of hotspots, a ranked list of these image blocks is determined based on the quantitation, using ImmunoRatio, of each block (Figure 4). “Step shifting” is illustrated in Figure 2 as well, while the black block moving from the yellow block indicates a ^1^/_4_ step shifting. ImmunoRatio provides quantitative image analysis of estrogen receptor (ER), progesterone receptor (PR), and Ki67 immunostained tissue sections [15]. In our software, the ImmunoRatio result is ranked and used to determine the hotspot areas.

The whole scanned image is segmented with NDPI splitter, as shown from the left upper image to the right upper image in Figure 4.

Based on the split images, we shift them by ^1^/_4_ of the side length, as shown from the right upper image to the bottom image in Figure 4. After the successful creation of JPEG images from the NDPI files, we adopt ImmunoRatio to calculate the IR% per block of the image, and rank the top 10 IR% image blocks.

### Adaptive step finding

**Figure 5:**
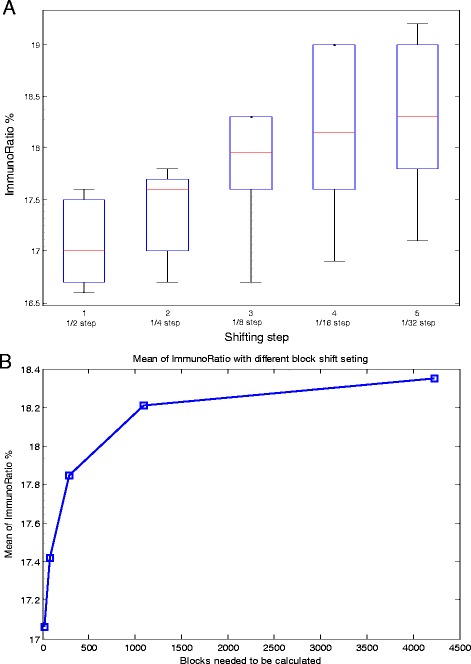
**The effect of (A) step size on ImmunoRatio % and (B) the blocks need to calculate these step sizes.** The average value as determined by ImmunoRatio (red line).

In this part, a smaller step finding procedure is applied to the top 10 images, regions of interest, obtained from the previous segmentation, ImmunoRatio and ranking procedure. The initial iteration uses ^1^/_2_ of shifting step from last iteration followed by more sensitive steps, such as 1/4 step (Figure 2) to precisely select the appropriate region of interest. Subsequently, the averaged top 10 ratios of current iteration are compared to the previous top ten ratios. The Step Finding procedure stops when the slope of ImmunoRatio to block number (as shown in Figure 5B) within a preset threshold of 0.01.

### Visualization and reporting

**Figure 6:**
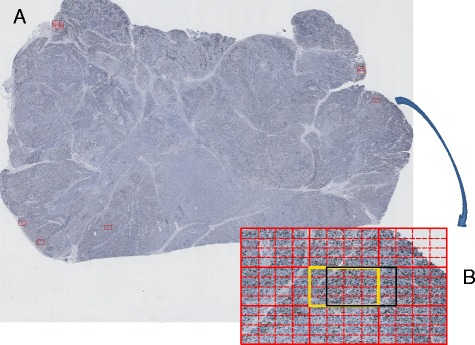
**Hotspot Reporting. (A)** The original image input for ASH analysis is overlaid with the hotspots (red rectangles). **(B)** The inset image shows the output from the adaptive step finding algorithm and the black box is the part that is displayed on the main image in **(A)** as a red rectangle.

In this part, we annotate the final top 10 regions in the original image and generate a report to list final top 10 ratios and their corresponding locations. Figure 6 shows an annotated image with Top 10 ImmunoRatio regions marked with red rectangles.

### Optimization of adaptive step selection

To determine the effect of step size of the performance of ASH, we calculated the averaged ImmunoRatio as the step size was decreased from ^1^/_2_ to ^1^/_32_ (Figure 5A). The averaged ImmunoRatio increases when step decreases from 17.07% to a maximum of 18.35% (Figure 5 and Table 1). The step size and its corresponding ImmunoRatio, block number, and processing time are indicated in Table 1. Figure 2 shows an example with ^1^/_4_ step shifting and its 81 (9×9) blocks. The more blocks are calculated, the more chances to obtain the block with higher ImmunoRatio. Decreasing the step size from ^1^/_2_ to ^1^/_32_ requires a non-linear increase in the number of blocks that must be calculated from 25 blocks up to 4225 blocks with an increase in average calculation time increase of >150 fold (i.e. from 25 seconds to about 1 hour per image) using a single core on an Intel Xeon X5650 CPU.

**Table 1:** **Optimization of ASH: The effect of step size on the performance of ASH was determined by as the average % ImmunoRatio (IR%), the number of blocks (# blocks) and the time in seconds to completion (Time) at decreasing step size intervals (Step size)**

**Step size**	**IR (%)**	**# blocks**	**Time (sec)**
1/2	17.06	25	25
1/4	17.42	81	82
1/8	17.85	289	291
1/16	18.25	1089	1097
1/32	18.35	4225	4254

### Validation of quantitative hotspot detection

Adaptive step finding has been utilized in engineering for many years [20] and extensively evaluated and validated by [21]. In [22], experimental evaluation demonstrates the effectiveness of the adaptive step size, while the adaptive step finding method applied in ASH had the same functionality. We have tested ASH in a set of >60 whole-slide digitally-scanned ACC images and in comparison with manual assessment labelling index assessment achieved a strong correlation (rho >0.8, p = 0) as calculated with Spearman rank order metric (publication in progress).

## Discussion

There are many commercial image analysis products such as AQUA [25], Genie (Aperio) [26], TissueStudio (Definiens) [27], InForm (PerkinElmer) [28] which are capable of high quality image processing and Ki67 quantitation, which are cited in other studies and are not freely available for comparative testing. Whilst there are several open source image analysis tools (e.g. ImageJ [29], ImmunRatio [17]) and multiple custom built in house applications (e.g. Seedlink [9]) and our requirements included that the applications be open source and that it could provide hotspot detection and quantitative Ki67 scoring in a desktop application. Thus, we developed ASH, an open source, open access, application using Galaxy-VM technology, to support histopathologists in determining the most active areas in proliferative rate within a slide based on Ki67 LI staining. Additionally since ASH was developed in a Galaxy environment the currently segmentation and quantitation methods can be easily supplemented or replaced, in the central ASH application (by the authors) or by a user (in their local ASH instance), with improved methods developed by other research teams.

We implemented an overlapping block creation method, Step Shifting, since NDPI splitter is only capable of splitting an image and not generating overlapping blocks and to support our Adaptive Step Finding method which has been utilised in multiple engineering projects over many years [20-22].

When we shift the image block by different steps, we can see that the averaged ImmunoRatio increases when step decreases. Therefore, we developed an adaptive step finding technique to obtain the tradeoff between hotspot detection resolution and processing time. Whilst the accuracy of the ImmunoRatio % per image block improves there is an increased cost for calculation time. Optimal calculation time to accuracy ratio occurs at ^1^/_16_ step size with ~1000 block based on the time to calculate one block is 1.0069 s based on a single core on an Intel Xeon X5650 processor.

Seedlink, a hybrid clustering method [9], that provides the users with automatic identification of hotspots is comparable to ASH with respect to usability and output. Seedlink requires a post-processing step to determine true hotspots from the false positive hotspots to ensure accurate determination of Ki67 whilst ASH provide a ranked set of regions for from which the user can include or reject as part of the quantitation of Ki67. Thus ASH simplifies the decision making process by integrating the visualization of the detected hotspots with the quantitation of detected hotspots as a single output in the Galaxy-VM GUI.

Since different types of colored pollutions and colour interferences sometimes cause trouble to the hotspot detection, Adobe photoshop or an alternative program enabling pathologists to delete parts of the scanned image i.e. artifacts created during slide production, will improve the accuracy of the hotspot detection. Whilst we have tested ASH in a training set it is clear that there are ‘inactive’ areas apparently with ‘low’ Ki67 Labelling index. Hence it is more prudent to compare automated selected hot spot areas versus hot spot areas as selected by pathologists and further studies are warranted to confirm our findings in a lager cohort.

Galaxy provides the user with a simple GUI to apply ASH using only standard web browser (see background, reference Galaxy). Galaxy provides the remote access for ASH, so people can benefit from the higher processing speed and larger storage space than a local computer. To ensure that ASH is available to individual researchers and/or pathologists as well as those who are supported by a bioinformatics team, we have implemented this Galaxy as a VMware-VM. The combination of Galaxy in a VM provides a multi-user environment in which users can analyse their images in a password protected user specific space, but with the additional functionality of Galaxy and the capability to share any of the data, analysis and results. The current Galaxy-VM has been implemented to run using 1 CPUs, but can be scaled up by resetting the VM once installed to run more CPUs (see project website for help documentation).

## Conclusions

We have developed ASH, an open source Galaxy virtual machine application designed for Ki67 LI hotspot detection support, aimed at both individual and large diagnostic laboratories who have little bioinformatics experience or support. ASH is designed to assist pathologists and accelerate the time-consuming Ki67 hotspot selection procedure, enhance the detection resolution and eventually lead to improved reproducible Ki67 LI reporting. Prior to image processing, pathologists should initially exclude with an interface tool various artifacts, such as tissue folds, intrinsic/extrinsic pigmentation (deposit artifacts), necrotic areas, *etc*. ASH delivers a ranked list of hotspots as a combination of images and quantitative values for each hotspot detected, based on the Adaptive step finding algorithm [20-22] developed as part of ASH. The selection of the step size is critical both from the point of view of computational efficiency and detection performance and although we have successfully tested ASH in a training set of whole-slide digitally-scanned ACC images, further studies are warranted in to confirm its efficiency with a larger ACC set.

## Availability and requirements

**Project name:** Automated Selection of Hotspots (ASH)

**Project home page:** http://bioinformatics.erasmusmc.nl/wiki/index.php/Automated_Selection_of_Hotspots which has a Galaxy VM instance of ASH.

**Operating system(s):** Windows, Linux (Ubuntu 12.04).

**Programming language:** C++, Bash, Java.

**Requirements:** VM ware player, Hamamatsu SDK, JAI 1.1.3, JAI Image IO 1.1, Ant, Deep Zoom.

License: GNU GPL version 3 [30].

### Ethical approval

These ACCs were assessed anonymously according to the Proper Secondary Use of Human Tissue code established by the Dutch Federation of Medical Scientific Societies (http://www.federa.org) and the Medical Ethical Committee of the Erasmus MC, Rotterdam, The Netherlands, approved the study.

## References

[1] Fassnacht M, Libé R, Kroiss M, Allolio B (2011). Adrenocortical carcinoma: a clinician’s update. Nat Rev Endocrinol.

[2] Beuschlein F, Obracay J, Saeger W, Kroiss M, Quinkler M, Lichtenauer UD, Deutschbein T, Ronchi CL, Willenberg H, Reisch N, Reincke M, Libe R, Baudin E, Bertherat JY, Haak H, Feelders RA, de Krijger R, Loli P, Terzolo M, Allolio B, Mueller H-H, Fassnacht M (2013). Prognostic value of histological markers in localized adrenocortical carcinoma after complete resection. Endocr Rev.

[3] Libé R, Borget I, Ronchi CL, Terzolo M, Haaf M, Laino F, Kerkhofs T, Corsini E, Tabarin A, Chabre O, de la Fouchardière C, Niccoli P, Caron P, Mannelli M, Haak H, Beuschlein F, Bertherat J, Berruti A, Fassnacht M, Baudin E (2013). Prognostic factors of advanced unresectable by stage III and IV ENS@T adrenocortical carcinomas (ACC). Endocr Abstr.

[4] Fassnacht M, Kroiss M, Allolio B (2013). Update in adrenocortical carcinoma. J Clin Endocrinol Metab.

[5] Dowsett M, Nielsen TO, A’Hern R, Bartlett J, Coombes RC, Cuzick J, Ellis M, Henry NL, Hugh JC, Lively T, McShane L, Paik S, Penault-Llorca F, Prudkin L, Regan M, Salter J, Sotiriou C, Smith IE, Viale G, Zujewski JA, Hayes DF (2011). Assessment of Ki67 in breast cancer: recommendations from the International Ki67 in breast cancer working group. J Natl Cancer Inst.

[6] Adsay V (2012). Ki67 labeling index in neuroendocrine tumors of the gastrointestinal and pancreatobiliary tract: to count or not to count is not the question, but rather how to count. Am J Surg Pathol.

[7] Mikami Y, Ueno T, Yoshimura K, Tsuda H, Kurosumi M, Masuda S, Horii R, Toi M, Sasano H: **Interobserver concordance of Ki67 labeling index in breast cancer. Japan breast cancer research group Ki67 ring study.** *Cancer Sci* 2013, doi: 10.1111/cas.12245 10.1111/cas.12245 PMC7656544 23905924

[8] Rindi G, Bordi C, La Rosa S, Solcia E, Delle Fave G (2011). Gastroenteropancreatic (neuro)endocrine neoplasms: the histology report. Gruppo Italiano Patologi Apparato Digerente (GIPAD); Società Italiana di Anatomia Patologica e Citopatologia Diagnostica/International Academy of Pathology, Italian division (SIAPEC/IAP). Dig Liver Dis.

[9] Lopez XM, Debeir O, Maris C, Rorive S, Roland I, Saerens M, Salmon I, Decaestecker C (2012). Clustering methods applied in the detection of Ki67 hot-spots in whole tumor slide images: an efficient way to characterize heterogeneous tissue-based biomarkers. Cytometry.

[10] Elie N, Plancoulaine B, Signolle JP, Herlin P (2003). A simple way of quantifying immunostained cell nuclei on the whole histologic section. Cytometry A.

[11] The Galaxy Project: **Online bioinformatics analysis for everyone** [http://galaxyproject.org]

[12] **VMware virtual machine** [http://www.vmware.com]

[13] Nocq J, Celton M, Gendron P, Lemieux S, Wilhelm BT (2013). Harnessing virtual machines to simplify next-generation DNA sequencing analysis. Bioinformatics.

[14] Deroulers C, Ameisen D, Badoual M, Gerin C, Granier A, Lartaud M (2013). Analyzing huge pathology images with open source software. Diagn Pathol.

[15] Tuominen VJ, Ruotoistenmaki S, Viitanen A, Jumppanen M, Isola J (2010). ImmunoRatio: a publicly available web application for quantitative image analysis of estrogen receptor (ER), progesterone receptor (PR), and Ki-67. Breast Cancer Res.

[16] NDPITools: **NDPI split download for Linux x86_64** [http://www.imnc.in2p3.fr/pagesperso/deroulers/software/ndpitools/download/ndpitools-1.6.5/lin64/ndpisplit]

[17] **ImmunoRatio home page** [jvsmicroscope.uta.fi/immunoratio]

[18] OpenCV: **OpenCV for Linux/Mac** [http://opencv.org]

[19] **Download Ubuntu Desktop** [http://www.ubuntu.com/download/desktop]

[20] Schumer MA, Steiglitz K (1968). Adaptive step size random search. IEEE Trans Automat Contr.

[21] White L, Day R (1971). An evaluation of adaptive step-size random search. Automat Control, IEEE Trans on.

[22] Mekuz N, Derpanis KG, Tsotsos JK (2006). Adaptive step size window matching for detection. IEEE ICIP.

[23] Goecks J, Nekrutenko A, Taylor J, Galaxy Team (2010). Galaxy: a comprehensive approach for supporting accessible, reproducible, and transparent computational research in the life science. Genome Biol.

[24] Smith JE, Nair R (2005). The architecture of virtual machines. Comput (IEEE Comput Soc).

[25] Camp RL, Chung GG, Rimm DL (2002). Automated subcellular localization and quantification of protein expression in tissue microarrays. Nat Med.

[26] **Aperio ePathology: Leica Biosystems, Nussloch, GmbH** [http://www.leicabiosystems.com/pathology-imaging/aperio-epathology/]

[27] **Tissue Studio 3.5: Definiens AG, Bernhard-Wicki-Straße 5, 80636 München, Germany** [http://tissuestudio.definiens.com/]

[28] **InForm: PerkinElmer, 940 Winter Street, Waltham, Massachusetts 02451, USA** [http://www.perkinelmer.com/CMSResources/Images/44-144380PRD_inForm.pdf]

[29] ImageJ: **Image Processing and Analysis in Java** [http://imagej.nih.gov/ij/docs/index.html]

[30] Free Software Foundation: **A Quick Guide to GPLv3 – GNU Project – Free Software Foundation (FSF)** [http://www.gnu.org/licenses/quick-guide-gplv3.html]

